# Clinical decision support using pseudo-notes from multiple streams of EHR data

**DOI:** 10.1038/s41746-025-01777-x

**Published:** 2025-07-02

**Authors:** Simon A. Lee, Sujay Jain, Alex Chen, Kyoka Ono, Arabdha Biswas, Ákos Rudas, Jennifer Fang, Jeffrey N. Chiang

**Affiliations:** 1 Department of Computational Medicine, University of California Los Angeles, Los Angeles, CA USA; 2 LA Health Services, Los Angeles, CA USA; 3 Department of Emergency Medicine, Harbor-UCLA Medical Center, Torrance, CA USA; 4 Department of Emergency Medicine, University of California Los Angeles, Los Angeles, CA USA; 5 Department of Neurosurgery, University of California Los Angeles, Los Angeles, CA USA

**Keywords:** Machine learning, Health care

## Abstract

Electronic health records (EHR) contain data from disparate sources, spanning various biological and temporal scales. In this work, we introduce the Multiple Embedding Model for EHR (MEME), a deep learning framework for clinical decision support that operates over heterogeneous EHR. MEME first converts tabular EHR into “pseudo-notes”, reducing the need for concept harmonization across EHR systems and allowing the use of any state-of-the-art, open source language foundation models. The model separately embeds EHR domains, then uses a self-attention mechanism to learn the contextual importance of these multiple embeddings. In a study of 400,019 emergency department visits, MEME successfully predicted emergency department disposition, discharge location, intensive care requirement, and mortality. It outperformed traditional machine learning models (Logistic Regression, Random Forest, XGBoost, MLP), EHR foundation models (EHR-shot, MC-BEC, MSEM), and GPT-4 prompting strategies. Due to text serialization, MEME also exhibited strong few-shot learning performance in an external, unstandardized EHR database.

## Introduction

In recent years, increased access to Electronic Health Records (EHR) has enabled the development and application of clinically relevant artificial intelligence (AI) and machine learning (ML). For example, both traditional and cutting-edge ML techniques have been harnessed to augment medical image interpretation^1^, drug discovery and delivery^2,3^, diagnosis^4,5^, and prognosis^6^, to name a few^7,8^. Due to the large variety of bespoke clinical applications built upon clinical health data, recent efforts have turned to developing generalist AI for healthcare^9,10^. Foundation models (FMs), the basis of large, generalist AI, are pre-trained on massive amounts of diverse data that exhibit adaptability and effectiveness across numerous domains^11^. These models have been shown to be adaptable to the healthcare setting, exhibiting state-of-the-art performance in multiple settings^12^.

The application of FMs to healthcare generally fits into one of two paradigms^13^. One approach augments widely-accessible large language models with clinical text (e.g., ClinicalBert^14^, MedPaLM^15^, GPT^11^, etc), taking advantage of the general reasoning capabilities of these models. For example, they have recently been able to generate discharge summaries from structured EHR without being trained on that particular task. However, continued adaptation of these models has been hampered by the fact that they are restricted to a text-based interface, making them incompatible with tabular EHR.

Another group of FMs is trained from scratch to operate upon sequences of discrete, structured items captured within the EHR (e.g., BEHRT^16^ and its variants^17^). EHR FMs have been shown to exhibit better predictive performance than bespoke ML models. However, there is substantially less data available to develop EHR FMs, which casts doubt on their general utility across diverse healthcare populations^13^. In addition to the relative lack of publicly available EHR for developing EHR FMs, a data standard is yet to be adopted that harmonizes tabular EHR across institutions^18–20^.

EHR are recorded in a variety of data types, including numerical, categorical, and free-text, which traditional ML has struggled to jointly process. These issues are partially addressed by EHR FMs, which can be configured to process categorical codes and continuous measurements^21^, but are limited by the need to harmonize these concepts. While EHR are commonly referred to and modeled by FMs as a single data type, these records span multiple biological scales and domains from laboratory measurements, to clinical interpretations and actions, to diagnostic codes. It is possible that this approach doesn’t capture the underlying distributions, given the high cardinality of EHR data and the relatively small amount of training data.

In this work, we present a modeling framework for EHR-based clinical decision support that overcomes key limitations in existing approaches. At the core of our framework is the concept of clinical pseudo-notes, a method for transforming tabular EHR data into text. This approach circumvents the need for explicit concept harmonization while serving as a natural language interface between structured EHR data and large language models. Using these pseudo-notes, we develop the Multiple Embedding Model for EHR (MEME), a novel architecture that processes distinct EHR concepts separately before integrating them through a self-attention mechanism, which acts as a form of feature extraction for EHR concepts prior to making final predictions.

To assess the effectiveness of MEME, we conduct comprehensive evaluations against both state-of-the-art EHR/biomedical FMs and traditional ML approaches. Our benchmarking includes MC-BEC^22^, a multimodal EHR model that predicts emergency department outcomes using a light GBM model operating over frozen RadBERT embeddings^23^; EHR-Shot^24^, a 141-million-parameter autoregressive FM, leveraging the CLMBR^25^ (Clinical Language Modeling-Based Representations) architecture to generate patient representations from coded medical event sequences; and Clinical Longformer^26^, a biomedical transformer model optimized for long-sequence processing, trained on biomedical text, capable of handling up to 4096 tokens, significantly exceeding the 512-token constraint of standard BERT models. Additionally, we compare MEME against traditional ML models such as Logistic Regression, XGBoost, and Multi-Layer Perceptrons, aligning with prior benchmarking methodologies in the emergency department benchmarking literature^27^.

Through a series of predictive tasks in the emergency department setting, we demonstrate that MEME consistently outperforms both traditional ML baselines and EHR FMs. Our findings underscore the advantages of leveraging multiple embeddings to represent structured EHR data, offering a robust and flexible paradigm for clinical decision support.

## Results

### Study design and cohort

This study was conducted retrospectively on datasets collected from the Beth Israel Deaconness Medical Center in Boston, USA^28^ (MIMIC) and the UCLA Health medical system in Los Angeles, USA (UCLA). From each database, deidentified EHR from emergency room visits were identified and extracted (MIMIC: *n* = 400,019, UCLA: *n* = 947,028) with additional details in Table 1. These were used to predict discharge and decompensation outcomes (see Methods) including Emergency Room disposition, discharge location (discharge), intensive care (ICU), and mortality as defined in Chen et al.^22^. The publicly available MIMIC database was used for model development and validation. We used a 70/15/15 split for the MIMIC Dataset which corresponds to sample sizes of 280,013/60,003/60,003 respectively treating each patient visit independently. Additionally for the ED Decompensation tasks we used the same split resulting in sample sizes of 110,605/23,701/23,701. Supplementary Fig. 1 further describes the inclusion and exclusion criteria for this study.

**Table 1:** Data Summary

Patient Encounters	MIMIC (*n* = 400,019)	UCLA (*n* = 947,028)
Median Age [IQR]	56 [35, 71]	42 [18, 66]
Male (*n*, %)	195,189 (48.8%)	518,532 (54.8%)
Race (*n*,%)		
White	228,123 (57.0%)	583,925 (61.7%)
African American	76,798 (19.2%)	139,497 (14.7%)
Asian	18,528 (4.7%)	83,329 (8.8%)
Other	76,570 (19.1%)	140,277 (14.8%)
Outcomes (*n*,%)		
Ed Disposition	158,007 (39.5%)	239,598 (25.3%)
Discharge Location	70,945 (44.9%)	91,287 (38.1%)
ICU	31,127 (19.7%)	37,616 (15.7%)
Mortality	4582 (2.9%)	7427 (3.1%)

Demographic and Clinical Characteristics of Patient Encounters at MIMIC and UCLA Hospitals.

The goal of our framework is to design a model that leverages off-the-shelf text models to represent EHR data effectively, addressing the challenges of variable-length inputs and the multistream nature of clinical records (e.g., triage information, medication info, vitals, etc). Streams of EHR data include, for example, diagnostic codes, prescription orders, and triage vitals, which represent separate biological and temporal scales. MEME processes each stream independently, embedding concepts separately to overcome token limit constraints (e.g., BERT’s 512-token limit), thus preserving the integrity of patient data without truncation (see “Methods”).

### Multiple embedding model for EHR (MEME)

**Fig. 1:**
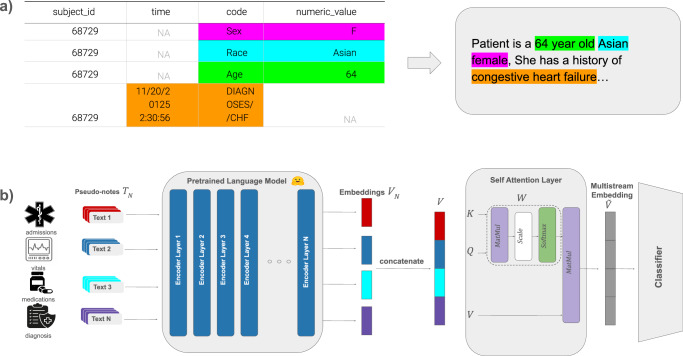
Overview of the pseudo-notes generation and multiple embedding model for Electronic Health Records (EHR). **a** Pseudo-notes generation. Tabular entries corresponding to different EHR concepts are separately constructed and integrated as fill-in features within predefined patient narratives. **b** Model architecture overview., which Multiple input streams representing distinct biological and temporal concepts within the EHR are encoded independently before being merged and processed through a self-attention layer. The resulting multistream embedding is then passed through a fully connected layer for downstream prediction.

EHR are heterogeneous datasets encompassing various biological and temporal scales, represented across multiple tables in categorical, numerical, and textual formats. Integrating these data types presents additional challenges in terms of data harmonization and standards adoption. Instead, we perform text-serialization^29^ in which tabular EHR are converted to text, which we refer to as clinical pseudo-notes (Fig. 1a).

While text generation with large language models has been explored^30–32^, persistent issues such as data hallucination pose significant challenges, as noted in ref. ^29^. Our approach uses a template approach (Fig. 1), in which structured data is inserted into a pre-configured template. This resembles the manner in which the majority of clinical text is generated (e.g., SmartPhrases/DotPhrases from the Epic/EMR system^33^). An example of generate pseudo-notes are available as Supplementary Fig. 2.

We process each stream independently, assigning a distinct embedding to each EHR data stream. This results in multiple paragraphs of clinical pseudo-notes, each containing a separate “domain” of EHR (diagnoses, encounter metainformation, medications, vitals, and information at triage). When data for an entire domain are unavailable for a patient, a default sentence indicating that the modality is missing is inserted (e.g., “Medications were not administered”). These embeddings are then concatenated and subjected to a self-attention layer, which synthesizes the entire patient context prior to decision-making (Fig. 1; see “Methods” for additional details). This self-attention layer serves as a feature extractor for EHR concepts which attend to certain components of the patient’s visit that inform making decisions.

Our approach assigns distinct embeddings to each EHR stream, which are then concatenated and processed through a self-attention layer to synthesize the patient representation for decision-making. Prior work, such as ExBEHRT^17^, along with our findings, demonstrates that this method improves performance by avoiding the truncation and ordering issues of single, heterogeneous embeddings. Figure 1 illustrates a schematic of the framework’s workflow.

In this study, embeddings for each pseudo-note paragraph are extracted using frozen language foundational models, resulting in high-dimensional vectors that capture various aspects of a patient’s medical history. In our work, we used MedBERT^34,35^ as the backbone encoder, but we highlight the general flexibility of our framework in using different encoders based on future developments (see “Multimodal embedding is compatible with evolving language models” section in the “Results”). These embeddings are then concatenated into a unified input vector for further processing. In the proceeding step, a self-attention layer analyzes the combined vector, capturing relationships between different medical concepts. The processed vector is then passed through a classifier to predict outcomes such as ED Disposition or Decompensation, with the model optimized using a tailored loss function.

This approach contrasts with existing efforts to develop foundational models for EHR representation^16,24,36^. We evaluated performance against representative foundational EHR models as well as baseline non-deep learning models trained from scratch^27^ on tabular data. Reference model comparisons were run within the MIMIC dataset only due to data harmonization issues with the institutional database and quantified in terms of the Area Under the Receiver Operating Characteristic Curve (AUROC), the Area Under Precision-Recall Curve (AUPRC), and F1 scores. 95% confidence intervals were generated for each metric by resampling the test set 1000 times.

### MEME vs EHR foundation models

EHR-specific FMs (EHR FMs) have been recently developed and have shown predictive capabilities across a variety of healthcare applications^13^ We selected the following reference EHR FMs as representatives of the approach: MC-BEC, EHR-SHOT, Clinical Longformer.

On the MIMIC validation set, MEME significantly outperformed EHR FMs in ED disposition as displayed in Tables 2–4. In the context of decompensation, MEME outperformed EHR FMs in all metrics when predicting ICU necessity and either outperformed or was statistically indistinguishable from EHR FMs when predicting mortality. We also show that by increasing the context window in the clinical longformer^26^ (512 tokens vs 1024 tokens), it does not necessarily result in better performance supporting the added benefit of our multiple embedding framework design.

**Table 2:** F1 scores benchmark on MIMIC dataset

Models/Tasks	ED Disposition	Discharge	ICU Requirement	Mortality
Logistic Regression	0.799 (0.025)	0.549 (0.033)	0.427 (0.036)	0.095 (0.026)
XGBoost	0.833 (0.022)	0.566 (0.030)	0.416 (0.057)	0.043 (0.019)
MLP	0.841 (0.010)	0.612 (0.013)	0.502 (0.019)	0.097 (0.023)
MC-BEC	0.912 (0.002)	0.653 (0.006)	0.545 (0.006)	0.127 (0.014)
EHR-Shot	0.874 (0.003)	0.691 (0.008)	0.560 (0.008)	0.036 (0.003)
Clinical Longformer	0.893 (0.002)	0.679 (0.007)	0.547 (0.008)	0.110 (0.009)
MEME	**0.943 (0.003)**	**0.698 (0.007)**	**0.572 (0.014)**	**0.137 (0.035)**

F1 Scores Table of EHR Foundation Models and traditional Machine Learning Benchmarking against our proposed MEME framework. Bold indicates the best performing model with underlines indicating second best. Values in the parentheses denote 95% confidence intervals.

**Table 3:** AUROC scores benchmark on MIMIC dataset

Models/Tasks	ED Disposition	Discharge	ICU Requirement	Mortality
Logistic Regression	0.863 (0.012)	0.852 (0.014)	0.807 (0.017)	0.768 (0.019)
XGBoost	0.909 (0.010)	**0.862 (0.016)**	**0.894 (0.016)**	0.845 (0.016)
MLP	0.871 (0.018)	0.802 (0.011)	0.767 (0.011)	0.786 (0.013)
MC-BEC	0.968 (0.02)	0.708 (0.006)	0.818 (0.014)	0.815 (0.006)
EHR-Shot	0.790 (0.031)	0.743 (0.007)	0.821 (0.018)	0.827 (0.009)
Clinical Longformer	0.888 (0.003)	0.739 (0.007)	0.819 (0.008)	0.811 (0.007)
MEME	**0.991 (0.001)**	0.799 (0.006)	0.870 (0.015)	**0.862** (**0.006)**

Area under the Receiver Operating Characteristic (AUROC) Scores Table of EHR Foundation Models and traditional Machine Learning Benchmarking against our proposed MEME framework. Bold indicates the best performing model with underlines indicating second best. Values in the parentheses denote 95% confidence intervals.

**Table 4:** AUPRC scores benchmark on MIMIC dataset

Models/Tasks	ED Disposition	Discharge	ICU Requirement	Mortality
Logistic Regression	0.874 (0.027)	0.628 (0.036)	0.618 (0.034)	0.051 (0.034)
XGBoost	0.912 (0.011)	0.642 (0.035)	0.630 (0.046)	0.128 (0.013)
MLP	0.866 (0.018)	0.630 (0.024)	0.581 (0.026)	0.077 (0.033)
MC-BEC	0.935 (0.003)	0.657 (0.009)	0.608 (0.009)	0.174 (0.025)
EHR-Shot	0.878 (0.007)	0.655 (0.012)	0.655 (0.017)	**0.246 (0.030)**
Clinical Longformer	0.902 (0.002)	0.634 (0.010)	0.642 (0.011)	0.211 (0.014)
MEME	**0.983 (0.002)**	**0.765 (0.008)**	**0.709 (0.012)**	0.243 (0.034)

Area under the Precision Recall Curve (AUPRC) Scores Table of EHR Foundation Models and traditional Machine Learning Benchmarking against our proposed MEME framework. Bold indicates the best performing model with underlines indicating second best. Values in the parentheses denote 95% confidence intervals.

### MEME vs traditional ML

We evaluated MEME against a logistic regression, xgboost, and neural network model^27^ operating over tabular EHR prior to pseudonote generation (Tables 2–4). Our objective in this benchmark was to test the juxtaposition between tabular and text-based representations on traditional ML models compared to our proposed method. MEME significantly outperformed these approaches in ED disposition. Evaluation on decompensation tasks were varied. The xgboost classifier outperformed MEME in terms of AUROC for discharge and ICU, and in terms of F1 for mortality. However, MEME significantly outperformed the same approaches in terms of AUPRC across all tasks. This could be due to differences in the incidence of these events, as discussed in refs. ^37–41^.

### Individual contributions of EHR concepts

MEME is composed of the combination of pseudo-notes as an interface between EHR and natural language LMs, and a multiple embedding approach in which EHR data domains are separately embedded. As shown above (Tables 2–4), the combination of these approaches achieves comparable or superior performance to alternative approaches for EHR modeling. We conducted the following studies to characterize the contribution of the multiple embedding approach over individual contributions from medical concepts.

MEME was referenced against a single modality embedding model (MSEM; see “Methods”), in which pseudo-notes were combined into one large text that produced a single heterogenous embedding. This significantly compromised predictive performance in all scenarios tested, highlighting the importance of embeddings for separate modalities to MEME’s performance^17^.

**Fig. 2:**
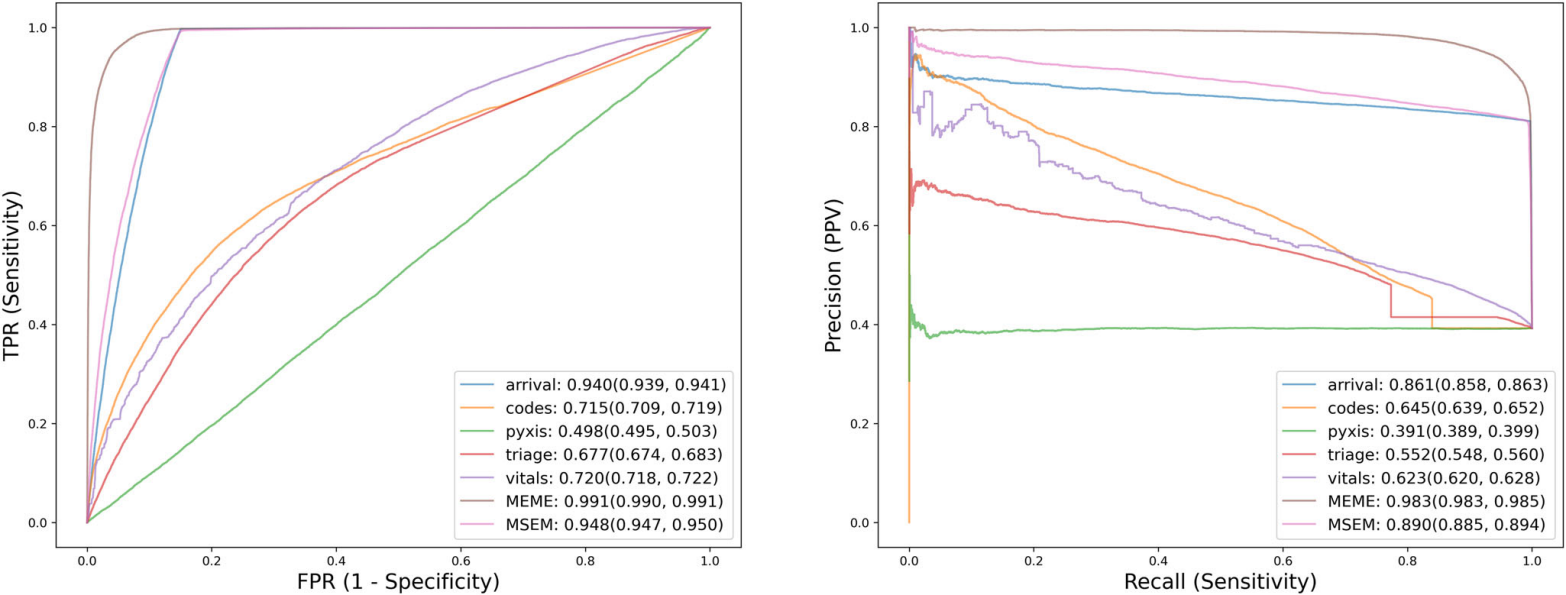
Model component study. Comparing Different Model Variants on Area under the Receiver Operating Characteristic (AUROC) and Precision-Recall Curves (AUPRC). The left panel displays AUROC curves for independent concept models versus a Multiconcept Single Embedding Model (MSEM) and our MEME model. The right panel illustrates AUPRC curves, depicting the precision-recall relationship for the same models. Model performance metrics (AUROC values) are annotated on both curves. It is evident that neither any single modality nor MSEM outperforms MEME.

A study was also conducted to characterize the individual contribution of different EHR input modalities, such that only pseudo-notes from one data category at a time could be considered. Again, no single modality on its own approached MEME’s performance (Fig. 2).

### MEME vs LLM prompting

**Fig. 3:**
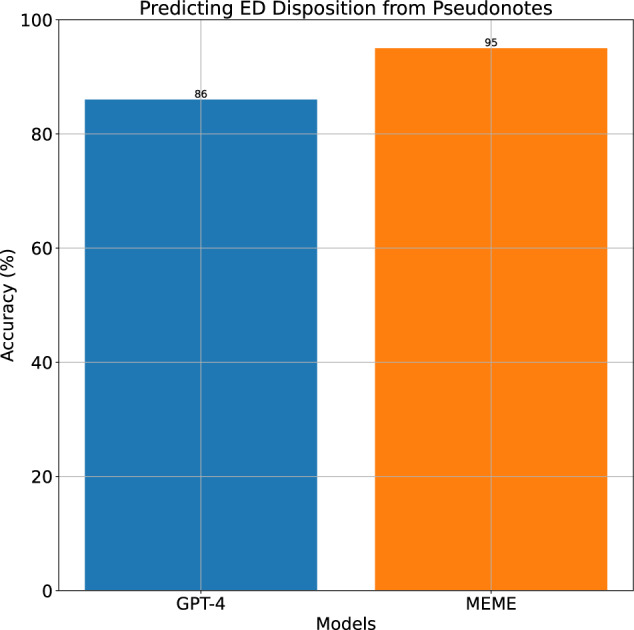
MEME vs instruction-tuned GPT-4. Predicting Emergency Department (ED) Disposition Using Pseudo-Notes Against Instruction-Tuned GPT-4. Accuracy of instruction-tuned GPT-4 and MEME in predicting ED disposition. While instruction tuning with 16 additional examples increased performance to 86%, it remained significantly below MEME’s 95% accuracy.

Given the emergent capabilities of generative AI models such as GPT-4, LLaMA-3, and Claude, we investigated the predictive performance of MEME relative to instruction-tuned (fine-tuned) large language models (Fig. 3). We tuned GPT-4 to predict ED disposition, and compared its performance against MEME’s classifier. Our prompt design for this task adhered to industry standards and followed conventional prompt engineering principles^42^. To assess whether GPT-4’s performance could be improved through task-specific adaptation, we applied instruction tuning by providing the model with 16 labeled examples before inference. On 100 randomly sampled pseudo-notes, instruction-tuned GPT-4 performed with 86% accuracy. Despite this, MEME still maintained a considerable performance advantage, achieving 95% accuracy, highlighting the substantial gap between training specialized classifiers and benchmarking them against high-capacity language models.

These results emphasize that training structured embedding-based classifiers remains superior to direct prompting approaches, even when instruction tuning is applied. Our findings align with recent studies^43^, which demonstrate that domain-specific models consistently outperform generalist AI models like GPT in structured clinical tasks, underscoring the continued need for EHR-specific ML models in healthcare applications.

### Multimodal embedding is compatible with evolving language models

**Fig. 4:**
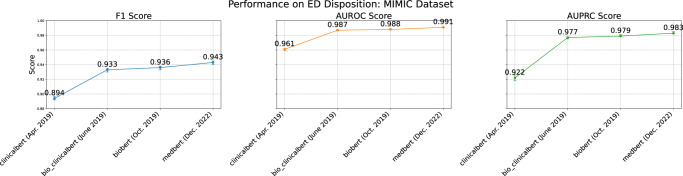
Performance generalization via few-shot learning. F1 score, AUROC (Area Under the Receiver Operating Characteristic Curve), and AUPRC (Area Under the Precision-Recall Curve) for few-shot learning models across multiple tasks as a function of increasing sample size. The plots illustrate steady improvements between 128 and 512 samples showcasing that this model can overcome OOD which is a current struggle of healthcare AI models.

The multimodal embedding approach in MEME is agnostic to the choice of the natural language model used to embed clinical pseudo-notes. To illustrate this flexibility, we evaluated MEME on the ED Disposition task using different pre-trained clinical language model backbones, including clinical BERT (April 2019), Bio_ClinicalBERT (June 2019), Bio BERT (October 2019), and Med BERT (2022). The results, presented in Fig. 4, demonstrate that as language model derivatives continue to evolve, they consistently yield improvements in predictive performance. This finding underscores that MEME is not dependent on any single language model but can seamlessly integrate any pre-trained encoder, allowing it to capitalize on advancements in clinical FMs. By showing a steady increase in performance metrics over time, Fig. 4 highlights MEME’s adaptability and extensibility, reinforcing its capacity to incorporate future innovations in language modeling to further enhance clinical decision support.

### Cross-institution generalization and adaptation

**Fig. 5:**
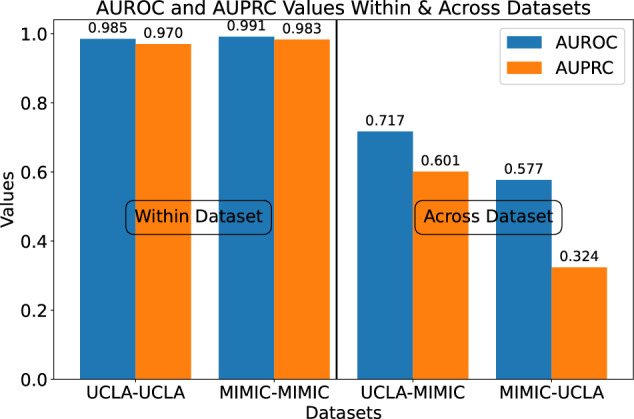
Performance of the ED disposition task across and within datasets. This figure illustrates the performance of MEME when evaluated within a single institution versus across different sites. MIMIC-MIMIC, UCLA-UCLA MEME is trained and evaluated using a train-test split within a single dataset. MIMIC-UCLA, UCLA-MIMIC MEME is trained using one dataset and evaluated on the other. A performance drop is observed when models trained only on one institution are directly applied to the other.

MEME exhibited strong performance within individual institutions but showed poor generalizability when directly applied across different sites (Fig. 5). This decline in performance is a well-documented challenge in healthcare, where models trained on data from one institution often fail to generalize due to variations in patient populations, clinical practices, and data collection methodologies. In our cross-site experiments, training on one hospital’s data and testing on another led to significant drops in F1, AUROC, and AUPRC scores, suggesting that dataset-specific characteristics remain a critical factor in model performance.

Although the pseudo-note approach was designed to mitigate variability across different EHR systems, our results indicate that differences in dataset composition still impact model generalizability. At a high level, patient demographics, prevalent conditions, and other differences reflected in the EHR concepts can vary significantly between institutions. The MIMIC dataset, sourced from hospitals in Boston, may reflect distinct population health trends compared to the UCLA dataset, representing patients from Los Angeles. These regional disparities influence not only the prevalence of certain conditions but also the patterns of medication prescriptions, diagnostic procedures, and clinical workflows, introducing distribution shifts that challenge out-of-distribution generalization. Additionally, differences in how data is recorded and structured between institutions may lead to subtle discrepancies in feature representation, further complicating cross-site adaptation.

However, few-shot learning offers a promising solution by enabling models to rapidly adapt to new environments with minimal data, improving generalization and robustness in the face of distribution shifts or out-of-distribution (OOD) data^44–46^. Many existing EHR FMs, while powerful, struggle with real-world applications due to their lack of interoperability with proprietary databases^11^. In contrast, the pseudo-notes approach used in MEME enhances interoperability, allowing for generalization across proprietary datasets when combined with few-shot learning, making it a more practical tool in diverse clinical settings.

To evaluate MEME’s adaptability, we tested its performance on an external population from the UCLA Health system. Fine-tuning MEME for the same ED disposition and decompensation tasks, we varied the number of local training samples from 2 to 1024. MEME achieved near-maximal performance (AUROC, AUPRC, and F1) between 128 and 512 samples, consistent with previous findings for EHR FMs like EHRShot^12^. This demonstrates MEME’s potential for real-world applications where rapid adaptation to new data is crucial.

## Discussion

In this work, we introduce MEME, a representation and decision-support framework for EHR. This approach uses pseudo-notes as an intuitive interface between structured electronic health data and foundational language models and adopts a multi-stream approach to encoding EHR data domains. The combination of these approaches results in comparable or superior performance compared with canonical and modern ML approaches across decision support tasks around Emergency Department disposition and decompensation.

Our study revealed that using multiple sources of EHR information independently appears to have significant results. We generally see that MEME outperforms all models with considerable improvement over EHR-shot, and the three standard methods on the ED disposition task. We also noticed that XGBoost performs better on two of the decompensation tasks in terms of the AUROC metric, but this could be nuanced due to class imbalance^37^. We notice more subtle improvements in all other metrics across all models.

**Fig. 6:**
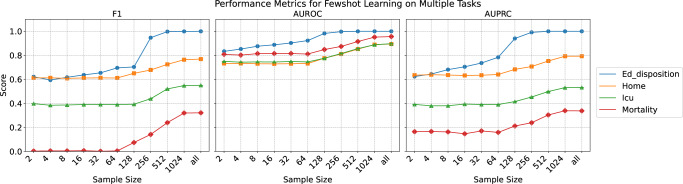
MEME backbone performance. Longitudinal Performance Metrics of MEME with Different Foundation Model Backbones for ED Disposition Prediction Using the MIMIC Dataset. Frozen, pre-trained language models were selected and used to generate pseudo-note embeddings and predict ED disposition using the MEME framework. F1 score, Area Under the Receiver Operating Characteristic Curve (AUROC), and Area Under the Precision-Recall Curve (AUPRC) improve over time as a function of improved backbone models.

We designed our model to be compatible with the pseudo-notes design, which encodes separate biological and temporal scales. To test the algorithmic design of MEME, we compared it with several baselines from ref. ^37^, ranging from traditional ML techniques to EHR FMs. Our study where we looked at individual contributions from EHR concepts revealed that the multi-stream approach, which integrates multiple concepts, significantly outperforms individual models and the Multi-stream Single Embedding Model (MSEM) (Fig. 6). This method enables MEME to represent each EHR concept with high fidelity and dynamically combine them for inference using self-attention. Our comparative studies further demonstrate that MEME surpasses both single-stream models and EHR FM alternatives, highlighting its superior capability in handling the multifaceted nature of healthcare data and supporting our design choices. Questions regarding context length were also studied where we compared our MEME approach against the clinical-longformer^26^ to motivate our framework design. We notice a considerable gap between these two methods, supporting our claims that different temporal and biological scales should be encoded separately instead of in a model with longer context length.

In addition to the performance advantages demonstrated by our experiments, MEME offers several qualitative benefits in terms of portability and extendibility. Unlike EHR-specific models such as BEHRT^16^, CHIRoN^47^, and EHR-shot^24^, which depend on evolving data standards and harmonization procedures for interoperability^18,48^, MEME utilizes a natural language approach that is extendible to any data that can be text-serialized (e.g. refs. ^29,49,50^), providing a straightforward interface for serializing both public and proprietary EHR systems. This approach is more easily adopted by institutions and can gracefully handle changes in coding standards, leveraging general reasoning capabilities and increasing the medical domain knowledge captured by existing and emerging foundation language models (Fig. 4). This framework not only promotes interoperability across diverse healthcare systems with varying protocols but also outperforms both EHR FMs and ML models which rely on harmonized structured formats, which are yet to be universally adopted.

One of the key motivations behind MEME’s design is the growing disparity between advancements in natural language processing (NLP) and the development of EHR FMs. Currently, language models trained on publicly available biomedical text—such as PubMed abstracts, textbooks, and clinical guidelines—demonstrate proficiency in learning and representing biomedical concepts, producing high-level representations that captured semantic relationships at the language level. In contrast, constructing effective representations for structured EHR data remained a challenge, particularly in high-dimensional tabular formats where traditional data harmonization methods struggled to maintain interpretability and generalizability.

Harmonizing structured EHR data across different institutions typically requires extensive preprocessing, such as mapping diagnoses, procedures, and medications into common ontologies. Canonical harmonization often introduces challenges due to differences in coding systems, institutional documentation practices, and evolving healthcare standards. For instance, ICD codes, which define diagnostic classifications, span thousands of distinct categories and typically require one-hot encoding to be processed by ML models. These methods result in sparse, high-dimensional feature spaces that can obscure intra-column relationships and limit model interpretability.

By converting tabular EHR data into pseudo-notes, MEME leverages pretrained NLP models and self-attention mechanisms to encode relationships between clinical concepts as natural language more effectively. Language models naturally capture contextual dependencies, allowing them to infer latent interactions between unstructured text elements without requiring explicit harmonization (Supplementary Figs. 5, 6). Unlike traditional tabular encoding methods, which often treat individual EHR features as independent variables, self-attention in transformer-based models can dynamically prioritize relevant clinical information across multiple temporal and biological scales.

Additionally, pseudo-notes provide a flexible and extendable interface that integrates seamlessly with existing and emerging foundation language models. While conventional ML models require domain-specific adjustments for different datasets, pseudo-notes allow MEME to remain agnostic to underlying data formats, making it compatible with any structured data that can be text-serialized. This design not only enhances interoperability across diverse healthcare systems but also ensures that MEME remains adaptable to future advancements in language model architectures, reinforcing its scalability and applicability in real-world clinical settings.

Healthcare AI models have often been criticized for failing to generalize across institutions^51^. We observed that MEME also displayed similar behavior due to little conceptual overlap between our two institutions resulting in distribution shifts that challenge out-of-distribution generalization (Supplementary Figs. 3, 4). However, it has been shown that foundational models are more efficient to adapt to new scenarios^15,52^. We found that MEME, using language-based embedding models, approached ceiling performance using between 128 and 512 training examples for ED Decision support tasks, which is comparable to the EHR FM EHR shot in the few-shot learning setting^12^. Coupled with the interoperability benefits of not requiring a data standard, this approach could be applied in settings where limited data annotations are available and the EHR are not recorded using a common data model.

The present study may have some limitations which include our inability to release our private institutional data, due to privacy restrictions and university policy. This highlights the significance of independent benchmarks, and underscores the necessity of external validation, for example benchmark datasets and tasks such as MC-BEC^22^. Additionally, our analysis was limited to a small set of hospital datasets and tasks from two sites, potentially not reflecting the full diversity of EHR systems. We did not investigate methods to harmonize different data schemas, which could affect the model’s adaptability across diverse healthcare settings.

In conclusion, we describe a decision-support modeling framework which interfaces between structured electronic health data and foundational language models. This approach is adaptable across a variety of settings and is compatible with evolving foundational models and may streamline the incorporation of modern AI into clinical decision support.

## Methods

### Data

Our study sources de-identified data from the publicly available Medical Information Mart for ICU (MIMIC)-IV v2.2 database and UCLA Health records. This analysis was deemed non-human subjects research by the local institutional review board (IRB) due to its retrospective and de-identified nature. We provide a detailed description of the EHR dataset’s composition, outlining the structured clinical and physiological information used in our predictive modeling.

The two datasets offer a structured representation of emergency department (ED) encounters, capturing categorical and quantitative measurements that have been used for both observational and predictive modeling studies^22,53,54^. Both datasets include triage vital signs, such as heart rate, blood pressure, respiratory rate, temperature, and oxygen saturation, recorded at the time of ED arrival to provide an initial physiological assessment. In addition, periodic vital sign measurements collected throughout the patient’s stay enable tracking of physiological changes over time.

The datasets further incorporate structured records of medications administered during the ED stay, including timestamps and dispensation details (pyxis). The medication reconciliation table (not available in the UCLA dataset) provides additional insights into pre-existing outpatient medication regimens, offering context on prior treatments. Discharge diagnoses, coded using ICD-9 and ICD-10 classifications, serve as a standardized clinical summary of patient conditions at the conclusion of the ED visit, facilitating downstream applications in outcome prediction and disease modeling.

Beyond clinical data, timestamped event records document key milestones during the ED stay, such as admission, transfer, and discharge. No outpatient records, prior hospitalization data, or additional longitudinal patient histories were included in our models.

MIMIC-IV ED^28^ is used for various downstream tasks, using EHR concepts such as arrival information, which captures patient demographics and means of arrival; triage, documenting patient vitals and complaints at arrival; medication reconciliation (medrecon), detailing prior and current medications; diagnostic codes (ICD-9/10) for diagnoses; and measurements throughout the ED stay, including patient vitals and medications from pyxis. Data across these modalities are linked via unique visit or hospital admission IDs (Hadm_id) and associated with all prediction labels.

De-identified patient information was extracted from the UCLA database^55^ to mirror the MIMIC-IV data modalities. Notably, this set lacked medication reconciliation (medrecon), and exhibited some concept variations due to different EHR system features. Our approach aims to make pseudo-notes across both databases closely resemble each other. Like MIMIC-IV, all concepts/streams in our UCLA data can be linked using a hospital admission ID and are also associated with all prediction labels.

In the MIMIC-IV database, we analyzed 400,019 unique visits, each associated with six modalities, contributing to a dataset size of ~2.4 million text paragraphs. For predicting ED disposition, we used the available data for training, validation, and testing with a set seed for reproducibility. For the decompensation prediction tasks, we utilized the subset of visits admitted to the hospital from the ED, resulting in a sample size of 158,010 patients. In the UCLA database, we analyzed a larger sample of 947,028 patients with five available modalities (excluding medrecon), resulting in ~4.75 million text paragraphs. All available data were used for the ED disposition task, and the 240,161 admitted patients were used for decompensation prediction. Further breakdowns can be found in our strobe diagrams in Table 1.

### Benchmark

This study focuses on binary and multilabel classification tasks related to Emergency Department (ED) disposition and decompensation, following definitions established in prior work, including the Multimodal Clinical Benchmark for Emergency Care (MC-BEC). To better align with clinical decision-making, we structure disposition prediction hierarchically. The first distinction is between patients who are discharged home and those who require hospital admission (ED Disposition). For patients who are admitted, we further assess their risk of decompensation as indicated by an upgrade of care or in-hospital mortality. By structuring the prediction task in this way, we explicitly model post-ED decision-making while also identifying cases where early warning signs of deterioration may inform clinical interventions. We evaluate the effectiveness of our multistream method in both single-label and multilabel classification tasks, benchmarking it against tabular-based and text-operating ML models to highlight the advantages of using a text-based, multiple-embedding strategy.

Specifically, the ED disposition prediction task aims to determine where a patient is sent following their ED visit based on EHR measurements recorded during their stay. This is framed as a binary classification problem, distinguishing between patients discharged home and those admitted to the hospital. Then, we aim to predict ED decompensation, which is defined as the acute physiological deterioration of a patient during their ED stay and the necessity of immediate medical intervention. While decompensation has many definitions across clinical contexts, we adopt the task defined by MC-BEC and assess three outcomes: whether a patient requires ICU admission, whether in-hospital mortality occurs, and/or whether a patient is discharged following hospital admission. These outcomes capture clinically relevant deterioration patterns that emerge during an ED visit and may require escalated interventions. We frame this problem as a multi label classification task in our evaluation.

To ensure a meaningful evaluation of decompensation, we restrict this analysis to only patients who were admitted to the hospital, distinguishing cases of acute physiological deterioration from routine ED visits that do not necessitate inpatient care.

### MEME

In the “Results” section, we introduced MEME. Here, in the “Methods” section, we provide a detailed explanation of how this framework progresses from processing pseudo-notes to generating predictions, outlining each step of the process comprehensively. This includes the transformation of raw data into structured embeddings, the application of self-attention mechanisms, and the integration of these embeddings into a predictive model, ensuring clarity at every stage of the pipeline.

In the initial step of our model, we aim to generate embeddings for each EHR concept by feeding tokenized data into our foundational models’ encoders, which produce rich, high-dimensional vector representations encapsulating various aspects of a patient’s medical history. We choose to freeze the encoder layers, focusing on the training parameters of the subsequent layers dedicated to the prediction task. After generating embeddings for all concepts, we concatenate them into a unified input vector for further processing. This procedure can be mathematically represented as follows: In the model’s first phase, modality-specific pseudo-notes are processed and structured into a tokenized format, denoted ${D}_{{tokenized}}$, (Eq. 1) which outlines a series of unique medical concepts or characteristics ${c}_{i}$ derived from a patient’s records. Each concept undergoes transformation via the FMs encoder into a high-dimensional vector $\vec{{v}_{i}}$, offering nuanced, context-rich portrayals of each EHR concept and capturing complex clinical information. These vectors are then unified into a comprehensive vector $\overrightarrow{{v}_{{concat}}}$ through concatenation, laying the groundwork for our multimodal patient embeddings (Eq. 2).

1 $$\overrightarrow{{v}_{i}}={\text{Foundation}}\,{\text{ Model}}({c}_{i})\forall {c}_{i}\in {\text{tokenized}}$$

2 $${\overrightarrow{V_{{concat}}}}={\text{Concatenate}}\left(\vec{{v}_{1}},\vec{{v}_{2}},\ldots ,\vec{{v}_{n}}\right)$$

In the second step of our network, we introduce a new use case of a self-attention layer^56^ designed to analyze the singular concatenated representation vector, $\overrightarrow{{V}_{{concat}}}$, as a unified entity. This approach arises from our intention to interpret aligned modalities collectively, rather than as separate entities, allowing the network to operate comprehensively on the entire vector (Eq. 3). It evaluates the relationships between elements within the vector, capturing patterns across different EHR concept vectors. The output from this layer is then directed through a fully connected layer, followed by a ReLU activation function (Eq. 4), before being fed into the final classifying layer for prediction (Eq. 5). This method, characterized by a unified analysis and attention-based processing, distinguishes our approach from traditional models and is pivotal to the enhanced predictive capabilities of our framework. This process is analogous to feature selection where the self-attention serves as a method for identifying what EHR concepts are relevant to the particular tasks. Figure 2 is evidence of this, where MEME found arrival information to be the most informative EHR concept related to admission related prediction tasks. Mathematically, this process involves transforming the input vector, $\overrightarrow{{V}_{{concat}}}$ into an attention vector $\overrightarrow{{V}_{{attention}}}$ using the self-attention mechanism, further processing it through a fully connected (FC) layer and a Rectified Linear Unit (ReLU) activation to obtain a refined feature vector $\vec{{V}_{{fc}}}$, as outlined below:

3 $$\overrightarrow{{V}_{{attention}}}={\text{Self}}\, {\text{Attention}}({\overrightarrow{V_{{concat}}}})$$

4 $$\overrightarrow{{V}_{{fc}}}={ReLU}({FC}(\overrightarrow{{V}_{{attention}}}))$$

5 $$\vec{z}={\text{Classifier}}\left(\vec{{V}_{{fc}}}\right)$$

The model leverages these refined features, $\vec{{V}_{{fc}}}$, in a classifier to produce logits $\vec{z}$, subsequently processed to predict probabilities for ED Disposition or ED Decompensation tasks. The classifier’s output is optimized by minimizing Cross Entropy Loss $L$, ensuring alignment of predicted probabilities ${\hat{y}}_{i}$ with true labels ${y}_{i}$. For multi-label tasks like ED

6 $$L=\mathop{\sum }\limits_{i=1}^{n}\mathop{\sum }\limits_{l=1}^{m}{BCE}\left(\sigma \left({z}_{i,l}\right){y}_{i,l}\right)$$

Decompensation, each logit $\vec{{z}_{i,l}}$ undergoes individual sigmoid activation $\sigma$, and the model’s training involves minimizing a tailored Cross Entropy Loss that aggregates binary cross-entropy losses across all labels for each observation, capturing the multi-label aspects of the data effectively (Eq. 6).

### EHR foundation model benchmarks

To systematically evaluate MEME against existing EHR FMs and compare their latent embeddings, we conduct a linear probing experiment. Since all benchmarked FMs produce a single fixed-size patient representation, we freeze these embeddings and train a simple linear classifier when a classifier is not specified in their respective literature to assess their predictive utility. This setup allows for a direct measurement of the effectiveness of their learned representations in downstream clinical tasks while ensuring consistency across models.

Our benchmarking includes MC-BEC, EHR-Shot, and Clinical Longformer—state-of-the-art EHR FMs that represent diverse approaches to encoding structured patient data. MC-BEC proposed a multimodal EHR model that encodes patient data using RadBERT embeddings before passing them through an LGBM classifier. Because it also leverages a pre-trained encoder pre-trained on radiology reports, we directly pass the pseudo-notes as input into this framework. EHR-Shot, a 141-million-parameter autoregressive FM, follows the CLMBR (Clinical Language Modeling-Based Representations) architecture to generate patient embeddings from sequences of coded medical events. For this model, we provide tabular data directly, as this is the expected input of an EHR FM for generating patient embeddings before training a linear classifier. Lastly, Clinical Longformer, a transformer model optimized for long-sequence processing, accommodates up to 4096 tokens, though we use 1024 tokens in our study. This significantly exceeds the 512-token constraint of standard BERT models, making it well-suited for handling long patient narratives. Since Clinical Longformer is an encoder-based model pre-trained on clinical text, we use pseudo-notes as input before training a linear classifier.

For a fair comparison, MEME follows the same linear probing setup but incorporates an additional self-attention mechanism before passing embeddings to the classifier. Unlike the benchmarked models, which produce a single embedding per patient, MEME generates multiple embeddings corresponding to distinct EHR concepts, using MedBERT as the backbone. The self-attention layer allows MEME to selectively attend to the most relevant EHR concepts—such as diagnoses and arrival information—before forming a unified patient representation. This design ensures that MEME’s final representation remains as comparable as possible to those generated by other FMs before classification, enabling a rigorous evaluation of the benefits of multiple embeddings in clinical prediction tasks.

### Multiconcept single embedding model (MSEM)

A natural question arises as to whether separate embeddings for different EHR concepts are necessary. To investigate this, we design an alternative approach analogous to MEME, where the entire patient narrative is encoded into a single heterogeneous embedding rather than maintaining distinct embeddings for different EHR concepts. This approach is conceptually similar to Clinical Longformer but is constrained to a sequence length of 512 tokens. We then train a linear classifier on top of these frozen heterogenous embeddings and evaluate its performance against MEME, as described in Fig. 6. This comparison allows us to assess the advantages of MEME’s multi-stream embedding strategy in contrast to a unified singular representation.

### Traditional machine learning benchmarks

To rigorously evaluate MEME against established methods, we benchmark its performance against a suite of traditional ML models that operate directly on tabular EHR data. These models serve as baselines to contrast tabular feature representations with our proposed text-based pseudo-note approach. The benchmarked models include Logistic Regression, XGBoost, and a baseline Multi-Layer Perceptron (MLP), each optimized with a grid search over the hyperparameters and these models were selected by prior literature^26^.

Logistic Regression, a widely used linear model, is trained with an L2 regularization penalty set to 1.0 and optimized using the SAGA solver, designed for large datasets in high-dimensional like clinical data. XGBoost, a gradient-boosting decision tree model, is configured with a learning rate of 0.05, a maximum depth of 6, and 1000 estimators, leveraging early stopping to prevent overfitting. The baseline MLP is designed to effectively process high-dimensional tabular EHR data. Rather than directly compressing the input into a small latent space, the model uses a gradual dimensionality reduction strategy with intermediate hidden layers. The architecture consists of five fully connected layers with 1024, 512, 256, 128, and 64 neurons, respectively, each followed by Batch Normalization and ReLU activation. To mitigate overfitting, dropout regularization (rate of 0.3) is applied to the first three layers, and weight decay (L2 regularization) is incorporated across all layers. The model is optimized using the Adam optimizer with an initial learning rate of 0.001, dynamically adjusted based on validation performance.

All of these models strictly process structured tabular data without incorporating text-based representations, enabling a direct comparison between conventional tabular feature engineering and our text based approach.

### Selecting optimal thresholds for F1, precision and recall

To select the optimal threshold for F1 and AUPRC (Area Under the Precision-Recall Curve) scores, we implemented a dynamic algorithm that samples thresholds from 0.00 to 1.00 in 1000 discrete steps. This approach allows us to identify the threshold that maximizes the F1 score and AUPRC by evaluating model performance at each point. The algorithm dynamically adjusts and evaluates precision, recall, and F1 at each threshold, selecting the one that strikes the best balance between precision and recall for the F1 score, while optimizing the trade-off between sensitivity and precision for AUPRC. By using such fine granularity in threshold selection, the model ensures that the chosen threshold is optimal for both metrics, leading to better prediction performance.

## Supplementary information

Lee_etal_Supplemental

## Data Availability

This study utilized publicly available datasets, including the MIMIC-IV Emergency Department (ED) dataset. These datasets can be accessed through their respective data portals subject to data use agreements (10.13026/07hj-2a80). UCLA EHR derived from the UCLA Health system cannot be shared publicly due to institutional restrictions and patient privacy considerations.

## Code availability

The code for preprocessing pseudo-notes as well as training MEME for classification, and multi-task learning models are available at the following GitHub repository: https://github.com/Simonlee711/MEME.
