# Supplementary material for: Clinical decision support using pseudo-notes from multiple streams of EHR data
Source: NPJ Digit Med. 2025 Jul 2;8:394. doi: 10.1038/s41746-025-01777-x (PMC12222764; doi:10.1038/s41746-025-01777-x)
Supplement: Supplementary file 1 — Lee_etal_Supplemental [file 41746_2025_1777_MOESM1_ESM.pdf]

Supplementary Information

Clinical decision support using pseudo-notes from multiple streams of EHR Data

Simon A. Lee, Sujay Jain, Alex Chen, Kyoka Ono, Arabdha Biswas, Akos Rudas,

Jennifer Fang, Jeffrey N. Chiang

Strobe Diagram and Outcome Breakdowns

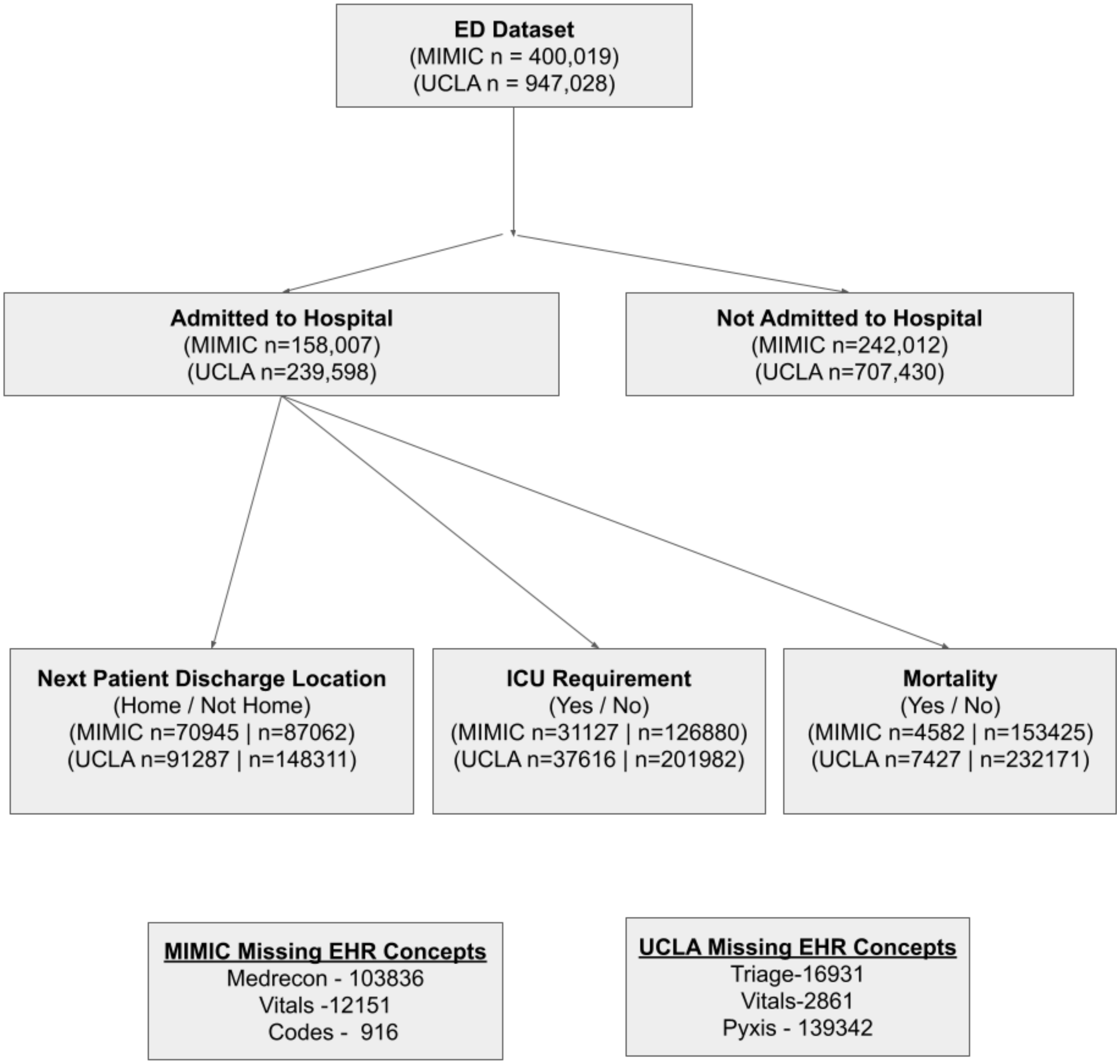

Supplementary Figure 1. STROBE Diagram for MIMIC and UCLA Cohorts. The diagram categorizes patients into those admitted to the hospital and those not admitted, with subsequent stratifications for discharge location, ICU requirement, and mortality. For each outcome of interest, we report the number of patients retained, along with corresponding label distributions and missingness counts. The dataset sizes indicate a significantly larger UCLA cohort compared to MIMIC. Additionally, the diagram highlights missing EHR concepts within each dataset, emphasizing gaps in documentation for medication reconciliation (Medrecon), vitals, and clinical codes in MIMIC, while UCLA has notable missing data in triage, vitals, and Pyxis medication administration records. Arrival Information:

The Patient is a 52 year old white female, arrived via ambulance at 2180-05-06 19:17:00. The patient's marital status is widowed. The patient's insurance is
Medicare. The patient's language is english.

Diagnostic Codes

The patient received the following diagnoses: ICD-9 code: [78959], other ascites. ICD-9 code: [07070], unspecified viral hepatitis c without hepatic coma. ICD-9 code: [5715], cirrhosis of liver nos. ICD-9 code: [v08], asymptomatic hiv infection.

Triage

At triage, the patient had the following measurements: temperature was 98.4, pulse was 70, respirations was 16, o2 saturation was 97, systolic blood pressure was 106, diastolic blood pressure was 63, pain was 0, chief complaint was abd pain, abdominal distention. Acuity score was 3.

Medrecon

The patient was previously taking the following medications: albuterol sulfate, asthma/copd therapy - beta 2-adrenergic agents, inhaled, short acting. peg 3350- electrolytes, laxative - saline/osmotic mixtures. nicotine, smoking deterrents - nicotine-type. spironolactone [aldactone], aldosterone receptor antagonists. emtricitabine-tenofovir [truvada], antiretroviral - nucleoside and nucleotide analog rtis combinations. raltegravir [isentress], antiretroviral - hiv-1 integrase strand transfer inhibitors. spironolactone [aldactone], diuretic - aldosterone receptor antagonist, non-selective. furosemide, diuretic - loop. ipratropium bromide [atrovent hfa], asthma/copd - anticholinergic agents, inhaled short acting. ergocalciferol (vitamin d2), vitamins - d derivatives.

Patient Vitals

The patient had the following vitals: At 2180-05-06 23:04:00, temperature was 97.7, pulse was 79, respirations was 16, o2 saturation was 98, systolic blood pressure was 107, diastolic blood pressure was 60, pain was 0.

Pyxis

The patient received the following medications: At 2180-08-05 22:29:00, morphine were administered. At 2180- 08-05 22:55:00, donnatol (elixir), aluminum-magnesium hydrox.-simet, aluminum-magnesium hydrox.-simet, ondansetron, ondansetron were administered.

Supplementary Figure 2. Pseudo-note example: A detailed example of a single patient visit pseudo-note. Each pseudo-note concept is generated independently using a text template that follows established guidelines in the literature literature[4]. The pseudo-note follows a structured format encompassing the following EHR concepts: patient demographics, diagnostic codes, triage information, medication reconciliation, vital signs, and administered treatments (pyxis).

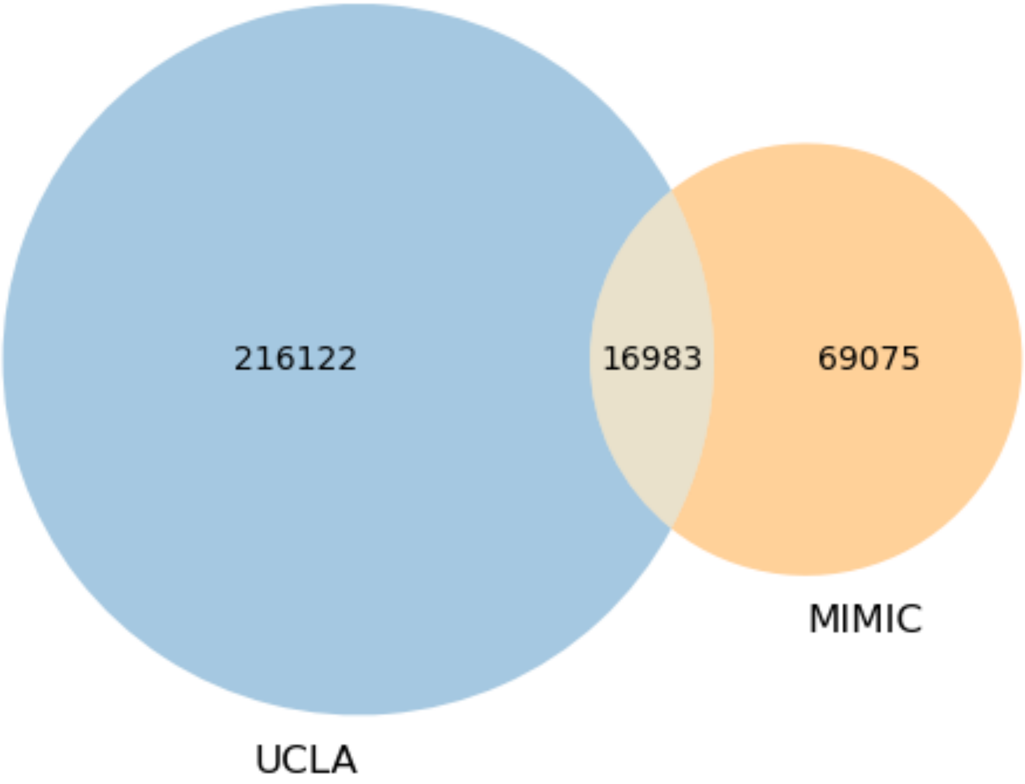

Supplementary Figure 3. Concept comparison between EHR datasets. The Venn Diagram quantifies the number of unique categorical concepts derived from the original tabular data, aggregated across all EHR data streams. There is little overlap in structured clinical representations, underscoring the heterogeneity inherent to multi-institutional EHR data and potential distribution shift.

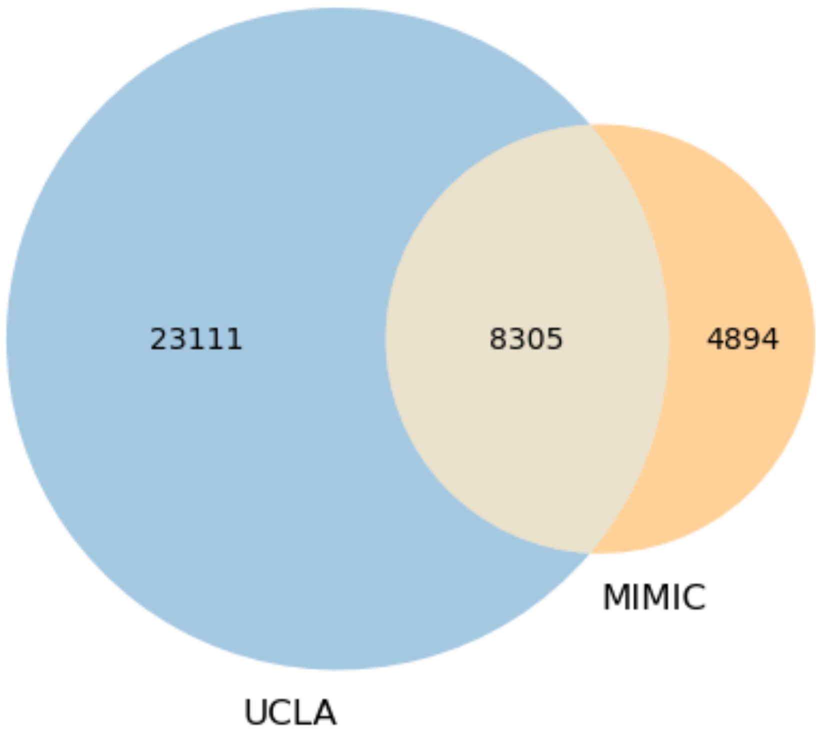

Supplementary Figure 4. ICD Code Comparison Between EHR Datasets. The Venn Diagram quantifies the number of unique diagnostic codes available across datasets. The limited overlap highlights the out-of-distribution issue in which treatment and recording protocols may differ across institutions, motivating alternative approaches to address dataset shift.

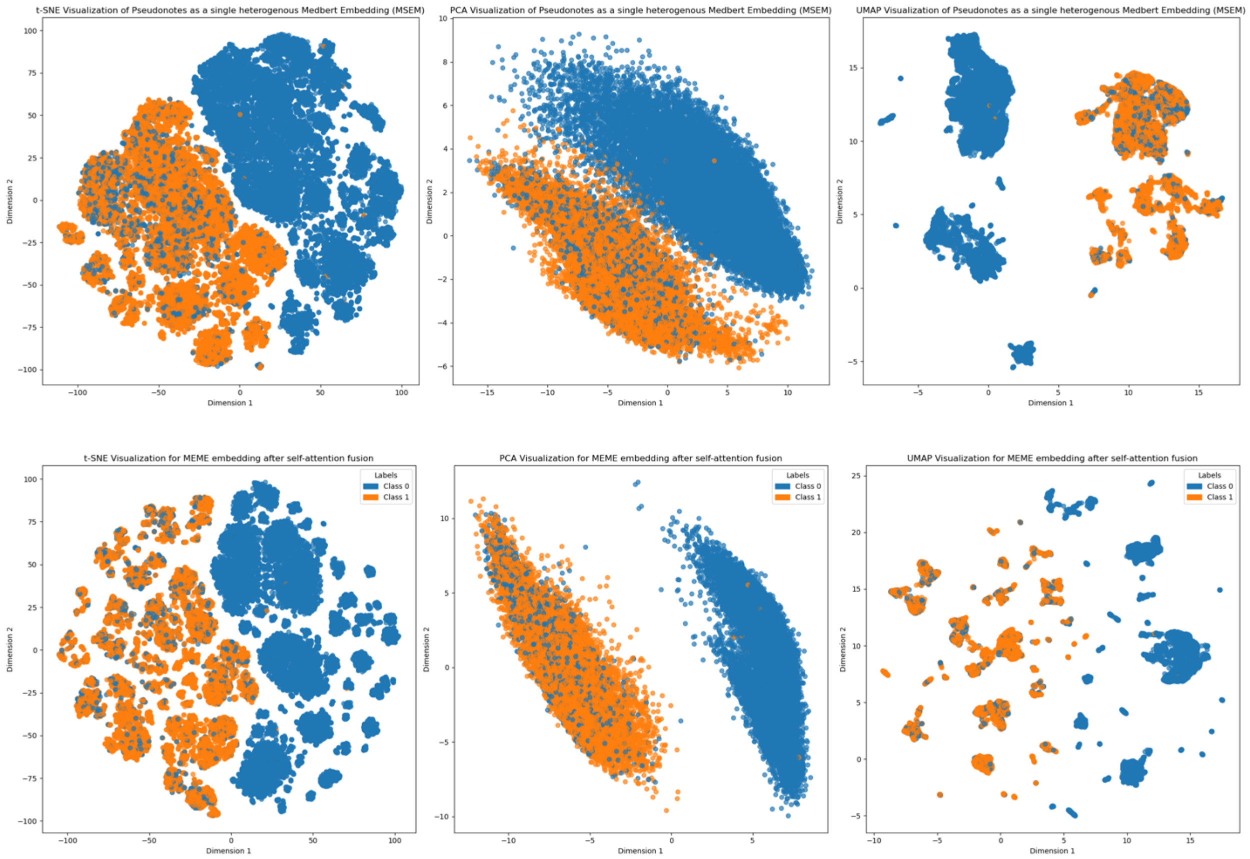

Supplementary Figure 5. **Latent Space Visualizations for MSEM and MEME.** Three 2D projections, t-SNE, PCA, and UMAP, compare the latent structure of the MSEM heterogeneous embeddings (top) against the MEME embeddings (bottom). These visualizations reveal that MEME captures clearer subcohort structure and achieves improved class separation within the latent space. Color encodes class labels from the ED Disposition prediction task, highlighting MEME's capacity to disentangle clinically meaningful groupings.

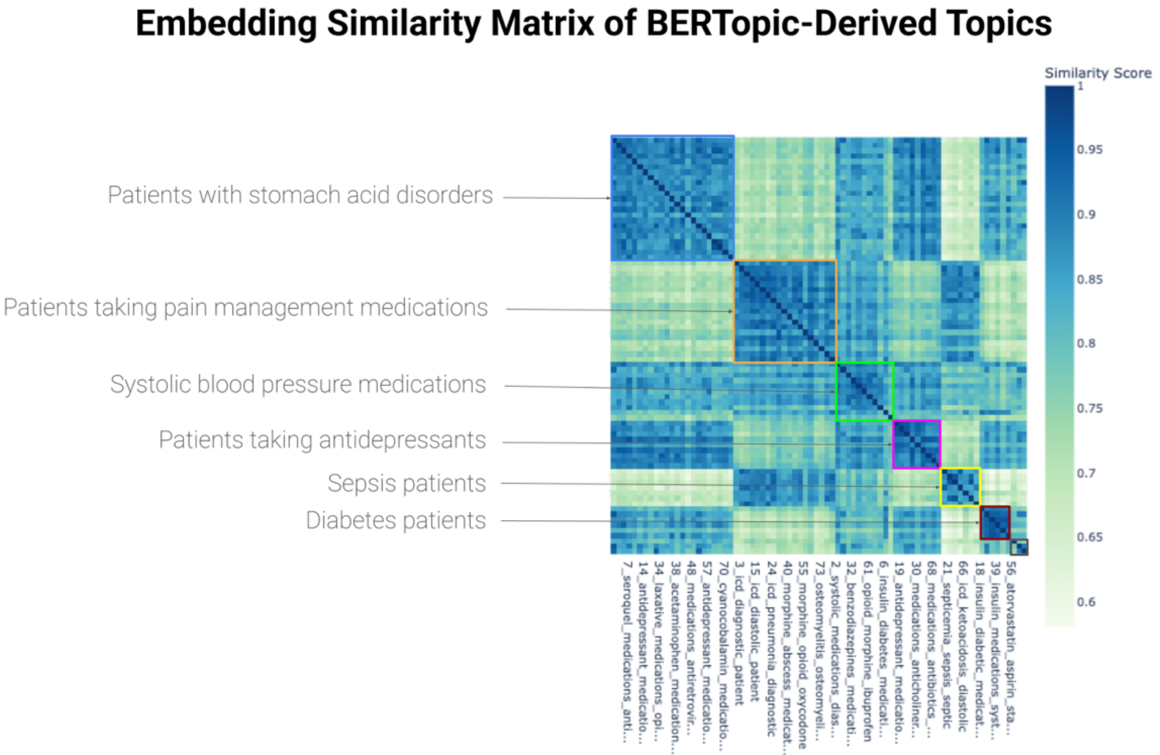

Supplementary Figure 6: BERTopic Analysis of MEME Latent Representations. BERTopic[56] reveals patient groups characterized by chief complaints and medication usage in a randomly sampled subset (n=40,001). The resulting similarity matrix reveals coherent groupings of patients, sorted by semantic proximity in the latent space, which provide interpretable insight into emergent clinical subphenotypes captured by the model in addition to the performance benefits of MEME.
